# A multispecific antibody confers pan-reactive SARS-CoV-2 neutralization and prevents immune escape

**DOI:** 10.1101/2022.07.29.502029

**Published:** 2022-08-04

**Authors:** John Misasi, Ronnie R. Wei, Lingshu Wang, Amarendra Pegu, Chih-Jen Wei, Olamide K. Oloniniyi, Tongqing Zhou, Bingchun Zhao, Misook Choe, Marika Boruszczak, Man Chen, Kwan Leung, Juan Li, Eun Sung Yang, Zhi-Yong Yang, Yi Zhang, Kevin Carlton, Darcy R. Harris, Vera B. Ivleva, Paula Lei, Cuiping Liu, Lindsay Longobardi, Adam S. Olia, Wei Shi, Jeremy J. Wolff, Jason Gall, Richard A. Koup, Peter D. Kwong, John R. Mascola, Gary J. Nabel, Nancy J. Sullivan

**Affiliations:** 1 Vaccine Research Center, National Institute of Allergy and Infectious Diseases, National Institutes of Health, Bethesda, MD 20892, USA.; 2 Modex Therapeutics Inc., an OPKO Health Company, Natick, MA 01760, USA.

## Abstract

Continued evolution of the SARS-CoV-2 spike poses a challenge to immune interventions. To develop antibodies that protect against evolving SARS-CoV-2 viruses, we combined antibodies that recognize different RBD sites to generate a trivalent antibody that potently neutralized all major variants, including the most recent Omicron lineages. Negative stain electron microscopy suggests that this multispecific achieves synergistic neutralization by engaging different epitopes in specific orientations that facilitate inter-spike binding. These interactions resulted in not only improved potency but also importantly prevented virus escape, a feature not seen with parental antibody cocktails or the most potent clinical antibody. Such multispecific antibodies simplify treatment, maximize coverage, decrease the likelihood of SARS-CoV-2 escape, and provide the basis for building universal SARS-CoV-2 antibody therapies that are more likely to maintain broad reactivity for future variants.

## One-Sentence Summary

SARS-CoV-2 multispecific antibodies expand neutralization breadth and potency, prevent immune escape, and simplify treatment.

**Figure 1.**
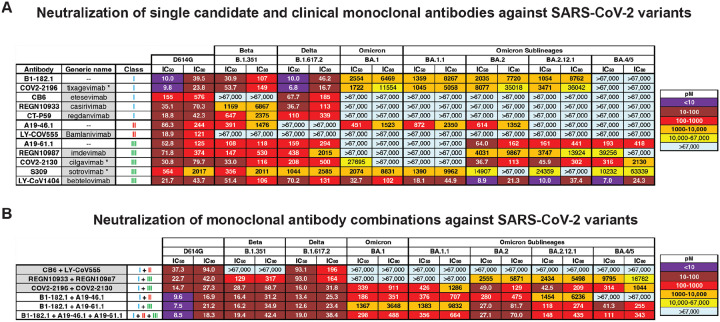
Neutralization of SARS-CoV-2 variants by monoclonal antibodies and antibody cocktails. Neutralization of candidate and expanded access monoclonal antibodies **(A)** and cocktails **(B)** against D614G and the indicated SARS-CoV-2 variants: Beta (B.1.351), Delta (B.1.617.2), Omicron (B.1.1.529 or BA.1) and Omicron sub-lineages. When appropriate, generic names are indicated. Generic names with * indicate the presence of Fc domain mutations in the clinical product that are not found in the experimental versions used in this paper. Class indicates the Barnes RBD classification(13): class I antibodies bind to the ACE2 binding site when RBD is in the up position; class II bind to the ACE2 binding site when RBD is in the up or down position; class III bind outside the ACE2 binding site when RBD is in the up or down position; and class IV bind outside of the ACE2 binding site when RBD is in the up position. Neutralization in pM is shown. Ranges are indicated with light blue (>67,000 pM), yellow (>10,000 to ≤67,000 pM), orange (>1,000 to ≤10,000 pM), red (>100 to ≤1,000 pM), maroon (>10 to ≤100 pM), and purple (≤10 pM).

The continued viral replication and transmission of viruses during human pandemics contribute to genetic evolution that can lead to increased pathogenesis and decreased sensitivity to host immunity and antivirals. For SARS-CoV-2, variations in the spike protein have resulted in the appearance of dozens of major variants of concern (VOC). VOCs such as Beta, Delta, Omicron and the newer Omicron sub-lineages contain several to dozens of amino acid variations in their spike protein that have been associated with decreased vaccine and therapeutic antibody efficacy (1). Among nine antibodies that have previously been, or are currently, approved as COVID-19 therapeutics (2–16), most have lost potency and/or breadth against evolving and variable circulating variants (Fig 1A). The emergence of the original Omicron (BA.1) VOC resulted in high-level resistance to several therapeutic antibodies, with only COV2–2196 (tixagevimab), S309 (sotrovimab) and LY-CoV1404 (bebtelovimab) remaining active (Fig 1A) (17–19). The subsequent BA.2, BA.2.12.1 and BA.4/5 variants showed additional changes in sensitivities: COV2–2196 became inactive against to BA.4/5, REGN10987 regained activity against BA.2 and BA.2.12.1, S309 lost potency against BA.2, BA.2.12.1 and BA.4/5 and COV2–2130 regained potency against BA.2, BA.2.12.1 and BA.4/5 (Fig 1A). This “cyclic” variation in potency is a key challenge to maintaining a portfolio of antibody-based therapies against COVID-19.

Among the clinical antibodies, only LY-CoV1404 has thus far maintained activity against all prior and current variants. However, as viral evolution continues, it is possible that resistant variants will develop to any single antibody. Therefore, there is a need to identify antibody therapeutics that can maintain activity in the face of evolving viral antigenic variation. We previously identified three monoclonal antibodies (mAb), B1–182.1, A19–46.1 and A19–61.1 (hereafter termed 182.1, 46.1 and 61.1), that target distinct receptor binding domain (RBD) epitopes and retain high potency and breath against most VOCs (17, 20): 182.1 displays subnanomolar potency against pre-Omicron VOCs (17, 20), including Beta and Delta (Fig 1A) and maintains potency to BA.1, BA.1.1, BA.2 and BA.2.12.1 that is similar to mAb S309 which showed clinical efficacy against BA.1 (21, 22); 61.1 has subnanomolar potency against Alpha, Beta and Delta variants but loses activity to Omicron BA.1 and BA.1.1, and then recovers neutralization activity against BA.2, BA.2.12.1 and BA.4/5 (17, 20) (Fig 1A); 46.1 is potently neutralizing against VOC that do not contain substitutions at L452, including Beta, Gamma, BA.1 and BA.1.1 (17, 20) (Fig 1A), but is inactive against BA.2.12.1 and BA.4/5 that have L452Q or L452R. Since these antibodies target distinct epitopes and show a non-overlapping pattern of VOC resistance, it suggested the possibility that a combination of these antibody specificities might provide neutralization breadth against longitudinal SARS-CoV-2 variants.

To test this hypothesis, we combined 182.1 with either 61.1 or 46.1 as two-mAb combinations or with both 61.1 and 46.1 as triple-mAb combination. These were tested for neutralization activity against Beta, Delta, Omicron and Omicron sublineages and results were compared with current clinical antibody cocktails. Consistent with previous reports, the therapeutic cocktails, CB6 + LY-CoV555 and REGN10933 + REGN10987, are unable to neutralize all VOCs (Fig 1B)(17, 23–25). In contrast, the combination of COV2–2196 + COV2–2130 (e.g., tixagevimab+cilgavimab) maintained subnanomolar potency across all variants albeit with some loss of potency (Fig 1B). As we previously showed (17), the combination of 182.1 + 46.1 provided synergistic neutralization against BA.1 (IC_50_ 186 pM) (Fig 1B) compared to the individual components (IC_50_ 2554 and 451 pM) (Fig 1A). In addition, we noted similarly potent synergistic neutralization against all Omicron sublineages except for BA.4/5; for which both mAbs lack activity (Fig 1). The combination of 182.1 + 61.1 neutralized all variants with IC_50_ values that were equivalent to or better than the parental antibodies (Fig 1), and the triple combination of 182.1 + 61.1 + 46.1 neutralized all variants with improved overall potency compared to the double combinations (Fig 1). These results show that combinations of 182.1, 46.1 and 61.1, targeting class I, II and III sites within the SARS-CoV-2 spike RBD, respectively, achieve potent neutralization across all prior and current VOCs.

**Figure 2.**
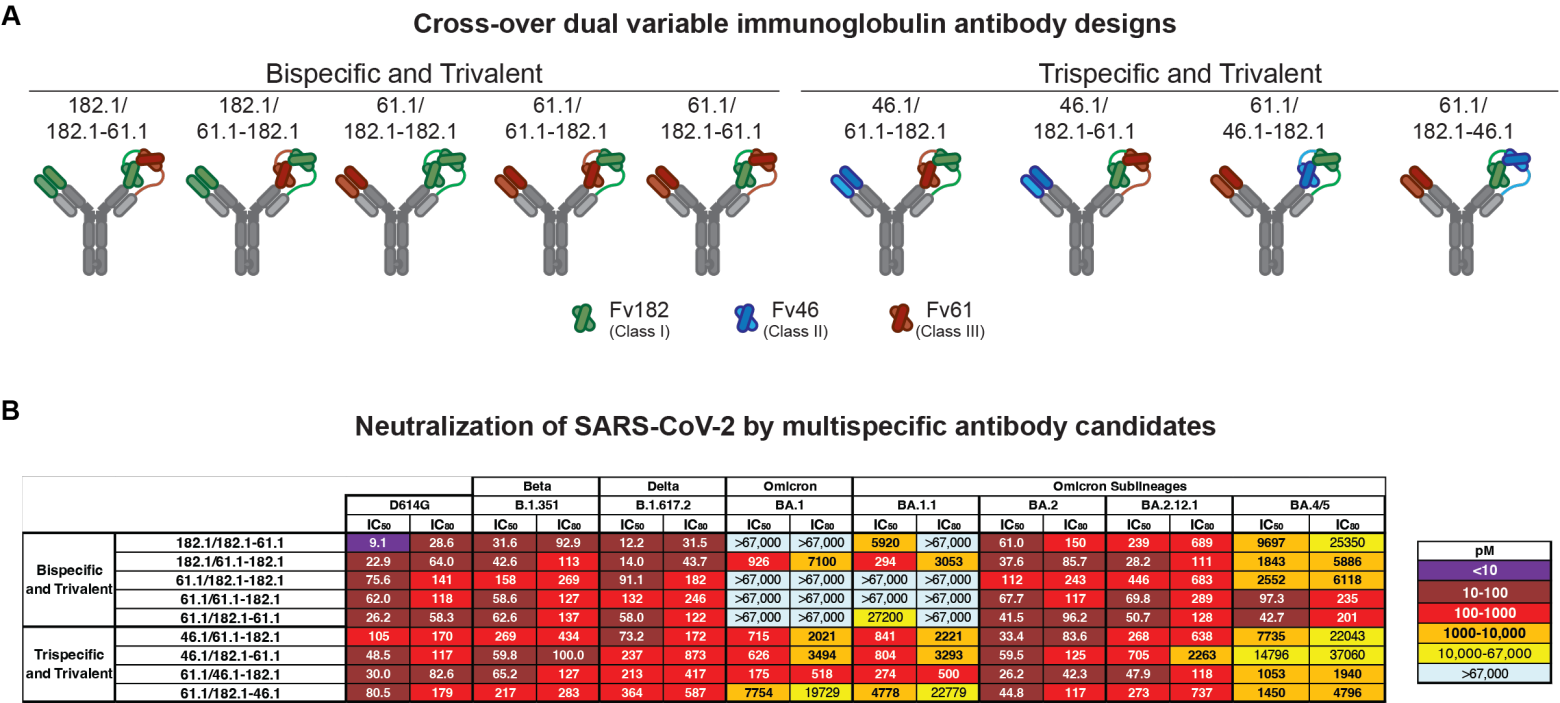
Selection of broadly reactive and potent SARS-CoV-2 monoclonal antibodies **A**. Cross-over dual variable (CODV) immunoglobulin antibody designs utilizing variable fragments (Fv) from B1–182.1 (Fv182.1, green), A19–46.1 (Fv46.1, blue) and A19–61.1 (Fv61.1, red). Fc regions contain “knob and hole” feature to increase yield and correct association of heavy chains. Five bispecific trivalent molecules (182.1/182.1–61.1, 182.1/61.1–182.1, 61.1/182.1–182.1, 61.1/61.1–182.1, 61.1/182.1–61.1) and four trispecific trivalent molecules (46.1/61.1–182.1, 46.1/182.1–61.1, 61.1/46.1–182.1, 61.1/182.1–46.1) were designed. **B**. Neutralization of candidate multispecific antibodies against D614G and the indicated SARS-CoV-2 variants, including Beta (B.1.351), Delta (B.1.617.2), Omicron (B.1.1.529 or BA.1) and Omicron sub-lineages. Neutralization in pM is shown. Ranges are indicated with light blue (>67,000 pM), yellow (>10,000 to ≤67,000 pM), orange (>1,000 to ≤10,000 pM), red (>100 to ≤1,000 pM), maroon (>10 to ≤100 pM), and purple (≤10 pM).

We previously developed recombinant trispecific antibodies against HIV-1 by combining arms from selected broadly neutralizing antibodies into a single molecule that showed unprecedented potency and breadth (26). These antibodies broadly neutralized >98% of circulating virus strains (26) and exerted antiviral effects in non-human primates while also mitigating the generation of viral immune escape (27). We hypothesized that multispecific antibodies could be designed against SARS-CoV-2 that similarly improve breadth and neutralization reactivity to cover known and evolving antigenic variation. We utilized the cross-over of dual variable (CODV) format comprising one arm heterodimerized with two variable fragment (Fv) domains and the second arm containing a single Fv domain (28). We designed nine trivalent anti-SARS-CoV-2 antibodies with bispecific (*i.e*., two antigenic targets) or trispecific (*i.e*., three antigenic targets) reactivity (Fig 2A, S1, Table S1). Five trivalent bispecific antibodies were generated: three containing two Fv182.1 (Fv182) and one Fv61.1 (Fv61) in different positions and two containing two Fv61 and one Fv182 in different positions (Fig2A, S1, Table S1). Four trivalent trispecific antibodies containing Fv182, Fv61 and Fv46.1 (Fv46) were designed to avoid placing Fv46 and Fv61 in the same CODV arm, since they were previously shown to compete with each other (20). To confirm the activity of each Fv component within the trivalent antibodies, we used ELISA to evaluate binding to SARS-CoV-2 RBD proteins containing mutations that specifically eliminate the binding of all but one Fv; specifically, these RBDs contained one or more of the previously identified knockout mutations for Fv182 (F486S), Fv46 (L452R) and Fv61 (K444E) (20) (Fig S2B). We found that each component Fv within the trivalent antibodies recognized its cognate epitope as expected, with equivalent binding to the wildtype RBD where the Fv epitopes were intact (Fig. S2C). As a further test, we assessed the ability of each antibody to neutralize pseudoviruses with spike protein point mutations that eliminated binding of a single Fv component and showed that each trivalent multispecific mAb maintained neutralization via the remaining Fvs (Fig S2D). Taken together, these findings verified that the Fvs within each multispecific antibody were functioning as expected.

We next evaluated the ability of the trivalent antibodies to neutralize the SARS-CoV-2 ancestral D614G, and the Beta, Delta, BA.1, BA.1.1, BA.2, BA.2.12.1 and BA.4/5 variants. For D614G, Beta and Delta variants, all of the multispecifics neutralized with subnanomolar IC_50_s (Fig 2B). Among the trivalent dual recognition (bispecific) antibodies that included 182.1 and 61.1 in different configurations, we observed that only the antibody with the 182.1/61.1–182.1 configuration maintained subnanomolar affinity against BA.1 and BA.1.1 (Fig 2B). These data indicate that for BA.1 and BA.1.1, the positioning of the Fv domain within a multispecific can impact neutralization potency. All of these bispecific antibodies displayed subnanomolar neutralization against BA.2 and BA.2.12.1 likely because, among the Fvs, the parental 61.1 antibody regains potent neutralization against these lineages. Interestingly, we found that having two copies of Fv61, regardless of position, significantly improved BA.4/5 neutralization >100-fold over other bispecifics antibodies containing a single Fv61 (Fig 2B). For the trispecific antibodies, all variants tested were neutralized with pico- to nanomolar potency. Against D614G, Beta and Delta variants IC_50_s ranged between 30 and 364 pM (Fig 2B). For BA.1, BA.1.1 and BA.4/5, neutralization by 61.1/46.1–182.1 occurred with IC_50_s of 175 pM, 274 pM and 1053 pM (Fig 2B), respectively, and notably for BA.1, with higher potency than the clinically effective antibody sotrovimab (IC_50_ 2074 pM) (Fig 1A). The 1–2 log higher potency of 61.1/46.1–182.1 for BA.1 and BA.1.1 compared to 61.1/182.1–46.1 suggests that the relative positions of 182.1 and 46.1 in the CODV arm are impacting on trispecific mAb potency. In contrast for BA.2 and BA.2.12.1, all four trispecific antibodies neutralized with IC_50_ between 26 and 705 pM, with 61.1/46.1–182.1 again having the highest potency (Fig 2B). These data indicate that a multispecific antibody with a precise configuration of Fv domains broadly neutralizes diverse strains (including strains that did not exist when the mAbs were made), suggesting that inter-epitope engagement increases antibody potency and breadth.

**Figure 3.**
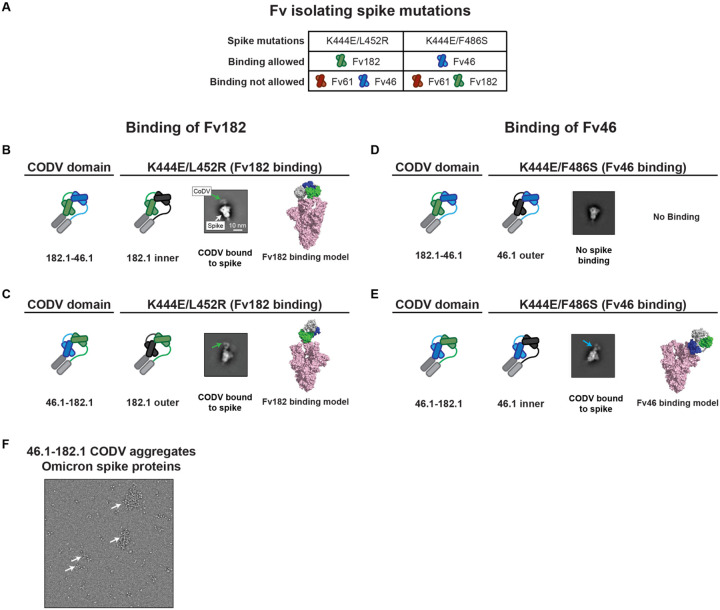
Identification of potential binding modes of CODV to SARS-CoV-2 spike by negative stain-electron microscopy **A**. Combination of RBD mutations on the SARS-CoV-2 spike designed to distinguish different binding modes of CODV. Mutations were made in spike in the spike trimer. The domains are colored green, blue, red and gray for Fv182.1, Fv46.1, Fv61.1 and constant domain, respectively. **B-E**. Evaluation of CODV Fv182 (B and C) binding to K444E/L452R or CODV Fv46 (D and E) binding to K444E/F486S. Shown at left in each panel is a schematic of the CODV domain being evaluated in the panel. The center-left subpanel indicates the Fv and position being evaluated (i.e., inner or outer). For clarity, the Fv domain that is not able to bind to the mutant is colored black in the subpanels. The center-right subpanel shows the negative stain electron micrograph class averages. Fv182 are indicated with a green arrow and Fv46 with a blue arrow. The white scale bar represents 10 nm in panel C and applies to each micrograph. The rightmost subpanel is a representative model of the binding mode observed in the micrograph. Panels B and C show that irrespective of position, both 182.1–46.1 and 46.1–182.1 are able to bind to spike protein that only allows binding by the Fv182. Panel D, shows that 182.1–46.1 (46.1 outer) is unable to bind spike protein that only allows binding by the Fv46.1. Panel E, shows that 46.1–182.1 (46.1 inner) is able to bind spike that only allows binding by Fv46. **F**. CODV 46.1–182.1 induced aggregation of the Omicron spike. Large clusters of aggregation were observed in the negative stain EM field, suggesting the CODV 46.1–182.1 can efficiently promote inter-spike crosslinking.

We note that the most potent and broad antibody, trispecific 61.1/46.1–182.1, contains the same component Fvs in its CODV arm as trispecific 61.1/182.1–46.1, yet neutralizes BA.1 and BA.1.1 15 to 40-fold better than 61.1/182.1–46.1 (Fig 2B). To better understand how differences in the positions of Fv46 and Fv182 in the CODV arm of these molecules led to differences in potency, we used negative stain-electron microscopy (NSEM) to compare the binding of purified CODV 46.1–182.1 or 182.1–46.1 to D614G spike trimer proteins containing mutations that eliminate binding to either Fv46 or Fv182 (Fig. 3A). Consistent with the 182 mAb epitope being at the distal tip of RBDs when they are in the up position, and thus more accessible for binding by Fv, the relative position of Fv182 within the CODV arm did not influence its ability to bind to the trimer (Fig 3B, C). However, for Fv46 we noted CODV position-dependent binding to spike trimers. In particular, when Fv46 is in the outer position (Fig 3D, **leftmost panels**), no NSEM binding classes were observed against K444E/F486S spike protein that should be recognized by Fv46 but is unable to bind Fv182 (Fig 3D, **rightmost panels**). Since CODV Fv46 binding to soluble RBD was not impacted (Fig S2C), these results suggests that the outer position the CODV is not compatible with binding of Fv46 to RBD domains contained within trimeric spike proteins. On the other hand, when Fv46 is in the inner position (Fig 3E, **leftmost panels**), NSEM class averages show that Fv46 binds K444E/F486S spike protein (Fig 3E, **center-right**). Representative models of the binding mode observed in the NSEM micrographs show that when Fv46 is in the outer position (Fig 3E, **right panel**), its angle of approach allows the CHCL1-Fv182 portions of the CODV to be positioned away from the spike trimer, thereby avoiding potential clashes with the spike trimer. Since NSEM of CODV 46.1–182.1 revealed that both Fv domains can bind spike trimers, we hypothesized that the CODV 46.1–182.1 alone would be sufficient to cross-link Omicron spike trimers. Indeed, NSEM micrographs revealed that aggregates were formed when CODV 46.1–182.1 was incubated with Omicron spike trimers (Fig 3E). Taken together, these results illustrate how in the context of spike trimers, both the location of epitopes within RBD and CODV position can impact binding, aggregative potential and neutralization. Specifically, Fvs with an angle of approach vertical to the trimer apex such as Fv182 are likely to have greater flexibility for positioning with trivalent mAb designs, likely due to freedom from steric constraints, and consistent with higher potency neutralization by this Fv. In contrast, Fv46 has a lateral or angled approach suggesting that antibodies that bind with this angle of approach may be subject to steric constraints that are revealed by position-dependence of the Fv for optimal engagement and neutralization.

**Figure 4.**
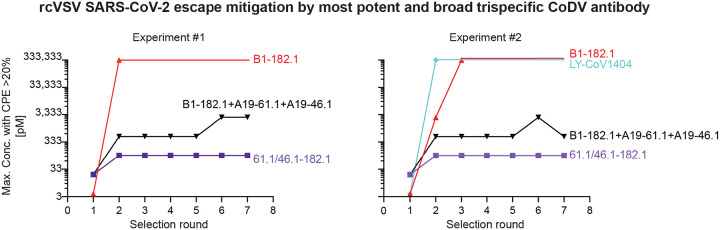
Mitigation of rcVSV escape by 61.1/46.1–182.1 and an antibody cocktail Replication competent VSV (rcVSV) bearing SARS-CoV-2 WA-1 spike protein was incubated with the indicated antibodies at 5-fold increasing concentrations (34 × 10^−3^ to 333,333 pM) and added to Vero cells. The appearance of viral growth, as indicated by the presence of >20% CPE, was determined after 3 days and supernatant from the highest antibody concentration with vial growth was passaged forward into a new selection round under the same conditions. Once viral growth appeared at 333,333 pM, the antibody was considered fully escaped and supernatant was no longer passaged forward. Data is graphed as the highest concentration of antibody at which viral growth was noted in each selection round. Each graph represents an independent experiment.

The perpetuation of the COVID-19 pandemic due to waves of infection by emerging VOC has demonstrated the impact of viral evolution and in particular, the impact of antigenic changes in the spike protein, on virus persistence. We previously showed that replication-competent vesicular stomatitis virus (rcVSV) pseudotyped with the SARS-CoV-2 spike can rapidly mutate to escape neutralization by the individual antibodies 182.1, 46.1 or 61.1 (20). We therefore sought to compare the potential for escape from the trivalent antibody with the broadest and most potent neutralization activity, 61.1/46.1–182.1, against the triple antibody cocktail or the individual mAbs, 182.1 and LY-CoV1404. Under conditions where 182.1 and LY-CoV1404 fully escaped antibody neutralization (i.e., >20% cytopathic effect at 333,333 pM) within 2–3 rounds of repeated infection *in vitro* (Fig 4), we found that an equal mixture of 182.1 + 61.1 + 46.1 slowed acquisition of the escaped phenotype, but gradually lost neutralization potency during 7 rounds of infection (Fig 4). For trispecific, 61.1/46.1–182.1, there was no observed escape in two independent experiments; though from round 1 to round 2 of replication there was a modest shift in the mAb concentration (107pM) required to maintain <20% CPE, in line with neutralization potency determinations that would not be expected to fully suppress viral growth (Fig 2B and 4). The ability of this trivalent mAb to mitigate neutralization escape corresponds with the observation that the CODV arm of 61.1/46.1–182.1 can also cross-link and aggregate spikes due to both Fv components being able to bind RBD (Fig 3C, E and F). Since the individual mAbs and mAb cocktail would not be expected to crosslink spikes. These results suggest that the potency and improved escape mitigation of 61.1/46.1–182.1 over the antibody cocktail is mediated by the ability to trivalent mAbs to engage and aggregate multiple trimers.

In this report, we developed trivalent bispecific and trispecific antibodies that target independent epitopes on the viral spike. These antibodies are highly potent and maintain breadth against VOCs with evolving patterns of antigenic variation, including the most recent Omicron sublineages. The combination of three antibody specificities in a precise orientation within one molecule ensured that the antibody could neutralize VOC with IC_50_s in a range similar to, or better than, clinically active antibodies, even when only one antibody Fv domain was active. In addition, the trispecific antibody mitigated virus escape *in vitro* under conditions where highly potent and broad single monoclonal antibodies could not, even when combined as cocktails. While the antibodies in this study were initially isolated prior to the emergence of Omicron, the strategy of rationally choosing antibody specificities that target distinct antigenic sites on spike, and that are differentially impacted by VOC mutations, allowed the generation of a broadly active molecule that neutralized future variants (unforeseen at the time of CODV design). Furthermore, the inclusion of multiple specificities on the same molecule has the potential of additive or synergistic binding and restriction of pathways for viral escape. Finally, the results in this report suggest the possibility that vaccine antigens targeting the functionally constrained epitopes contained in this trispecific antibody might increase breadth and potency against current and future variants using a single protein with simplified clinical development.

## Supplementary Material

Supplement 1

## Data Availability

All data is available in the main text or the supplementary materials.
