# Supplementary material for: A multispecific antibody confers pan-reactive SARS-CoV-2 neutralization and prevents immune escape
Source: bioRxiv. 2022 Aug 4:2022.07.29.502029. Preprint. [Version 2] doi: 10.1101/2022.07.29.502029 (PMC9387125; doi:10.1101/2022.07.29.502029)
Supplement: Supplement 1 [file media-1.pdf]

Supplementary Materials for

A multispecific antibody confers pan-reactive SARS-CoV-2 neutralization and prevents immune escape

Authors:
John Misasi[1 #], Ronnie R. Wei[2#], Lingshu Wang[1 #], Amarendra Pegu[1 #], Chih-Jen Wei[2#], Olamide K. Oloniniyi[1], Tongqing Zhou[1], Bingchun Zhao[1], Misook Choe[1], Marika Boruszczak[1], Man Chen[1], Kwan Leung[1], Juan Li[2], Eun Sung Yang[1], Zhi-Yong Yang[2], Yi Zhang[1], Kevin Carlton[1], Darcy R. Harris[1], Vera B. Ivleva[1], Paula Lei[1], Cuiping Liu[1], Lindsay Longobardi[1], Adam S. Olia[1], Wei Shi[1], Jeremy J. Wolff[1], Jason Gall[1], Richard A. Koup[1], Peter D. Kwong[1], John R. Mascola[1+], Gary J. Nabel[2*+], Nancy J. Sullivan[1*+]

Affiliations:

[1]Vaccine Research Center, National Institute of Allergy and Infectious Diseases, National Institutes of Health, Bethesda, MD 20892, USA.

[2]Modex Therapeutics, Natick, MA 01760, USA.

Equal contribution: #, +

*Corresponding authors: Nancy J Sullivan, Gary Nabel

Email: njsull@mail.nih.gov, gary.nabel@modextx.com

This PDF file includes:

Materials and Methods

Figs. S1 to S2

Tables S1

Materials and Methods

Design of trispecific antibodies

The trispecific antibody format follows the previously described design configuration (*26*). Briefly, CODV-Ig bispecific antibody arm (*29*) is heterodimerized with a conventional antibody arm using knob-in-hole (*30*) mutations in the CH3 domain of IgG1 Fc. Specifically, three classes of anti-SARS-CoV-2 neutralizing antibodies targeting RBDs B1-182.1 (class I), A19-46.1 (class II), and A19-61.1 (class III) were tested for possible combinations including each antibody Fv orientation and copy number to achieve bi- or tri-specificity. These combinations were tested for best neutralizing breadth/potency. In addition, "Knob" (S354C/T366W), "Hole" (Y349C /T366S/L368A/Y407V) (*30*), and "LS" (M428L/N434S) (*31*) mutations were engineered into CH3 of the monospecific, bi- or trispecific Fc region.

Synthesis, cloning and expression of multispecific antibodies

After design of the amino acid sequences for each multispecific antibody, the four genes for each multispecific antibody were synthesized using human preferred codons (GenScript) and cloned into eukaryotic expression vectors. For each multispecific antibody expression, equal amounts of the 4 plasmid DNAs were transfected into Expi293 cells (Life Technology) using Expi293 transfection reagent (Life Technology) as previously reported (*26*). The transfected cells were cultured in shaker incubator at 120 rpm, 37 °C, 9% CO2 for 4~5 days. Culture supernatants were harvested and filtered, the multispecific antibodies were purified over a Protein A (GE Health Science) column. Each multispecific antibody was eluted with IgG elution buffer (Pierce), immediately buffer exchanged with PBS and concentrated using Centricon Plus-70 (Millipore Sigma) membrane filter unit. After concentration, each multispecific antibody was applied to a Superdex 200 16/600 size exclusion column (Cytiva) to remove aggregates and different species in the preparation. The fractions were then analyzed on reduced and non-reduced SDS-PAGE to identify the fractions that contained the monomeric multispecific antibody before combining them. The pooled fractions were then further concentrated, aliquoted and analyzed by SDS-PAGE as well as an analytical SEC column (Superdex 200 16/600) to verify purity. Molecular weight, extinction coefficient and predicted pI were determined using Geneious Prime (Biomatters Ltd.)

To make the CODV from multispecific IgG, the FabALACTICA protease (Genovis) was used to digest the IgG for 16 hrs at room temperature. The digestion mixture was then incubated with protein A resin to remove Fc and undigested IgG, the flowthrough and PBS wash of the protein A column that contained the Fab and CODV fragments was collected, concentrated and further purified with size exclusion column (Superose 6 10/300, Cytiva)

Full-length S constructs

Codon optimized cDNAs encoding full-length S from SARS-CoV-2 (GenBank ID: QHD43416.1) were synthesized, cloned into the mammalian expression vector VRC8400 (*32*, *33*) and confirmed by sequencing. S containing D614G amino acid change was generated using the wt S sequence. Other variants were made by mutagenesis using QuickChange lightning Multi Site-Directed Mutagenesis Kit (cat # 210515, Agilent) or via synthesis and cloning (Genscript) as previously reported (*20*, *34*). The S variants tested are B.1.351 (L18F, D80A, D215G, (L242-244)del, R246I, K417N, E484K, N501Y, A701V), P.1 (L18F, T20N, P26S, D138Y, R190S, K417T,E484K, N501Y, D614G, H655Y, T1027I, V1176F), B.1.1.7 (H69del, V70del, Y144del, N501Y, A570D,D614G, P681H, T716I, S982A, D1118H), B.1.617.2 (T19R, G142D, E156del, F157del, R158G, L452R,T478K, D614G, P681R, D950N), B.1.1.529 or BA.1 (A67V,H69del, V70del, T95I, G142D, V143del, Y144del,Y145del, N211del, L212I, ins214EPE, G339D,S371L, S373P, S375F, K417N, N440K, G446S,S477N, T478K, E484A, Q493R, G496S, Q498R,N501Y, Y505H, T547K, D614G, H655Y, N679K,P681H, N764K, D796Y, N856K, Q954H, N969K,L981F) and BA.2 (T19I, L24-, P25-, P26-, A27S, G142D, V213G, G339D, S371F, S373P, S375F, T376A, D405N, R408S, K417N, N440K, S477N, T478K, E484A, Q493R, Q498R, N501Y, Y505H, D614G, H655Y, N679K, P681H, N764K, D796Y, Q954H, N969K). These full-length S plasmids were used for pseudovirus neutralization assays.

Pseudovirus neutralization assay

S-containing lentiviral pseudovirions were produced by co-transfection of packaging plasmid pCMVdR8.2, transducing plasmid pHR' CMV-Luc, a TMPRSS2 plasmid and S plasmids from SARS-CoV-2 variants into 293T cells using Lipofectamine 3000 transfection reagent (L3000-001, ThermoFisher Scientific, Asheville, NC) (*35*, *36*). 293T-ACE2 cells (provided by Dr. Michael Farzan) were plated into 96-well white/black Isoplates (PerkinElmer, Waltham, MA) at 7,500 cells per well the day before infection of SARS CoV-2 pseudovirus. Serial dilutions of mAbs were mixed with titrated pseudovirus, incubated for 45 minutes at 37°C and added to cells in triplicate. Following 2 h of incubation, wells were replenished with 150 ml of fresh media. Cells were lysed 72 h later, and luciferase activity was measured with MicroBeta (Perking Elmer). Percent neutralization and neutralization IC50s, IC80s were calculated using GraphPad Prism 8.0.2.

Antibody binding to RBD mutation proteins by ELISA

MaxiSorp Immuno plates (Thermo Fisher) plates were coated with 1 µg/ml of SARS-CoV-2 WA-1 RBD or RBD with single, double or triples mutations (F486S, E444K, L452R, F486S/E444K, F486S/L452R and F486S/E444K/L452R) in PBS at 4 °C overnight. After standard washes and blocking, plates were incubated with serial dilutions of antibody for one hour at room temperature. Anti-human IgG Fc γ-specific horseradish peroxidase conjugates (Jackson Laboratory) was used to detect binding of antibody to the RBD proteins. The plates were then washed and developed with 3,5,3′5′-tetramethylbenzidine (TMB) (KPL, Gaithersburg, MD). After stopped with 1N H2SO4 (Fisher), OD 450 nM was read with a SpectraMax Plus microplate reader (Molecular Device).

rcVSV antibody escape assay

Selection of virus escape variants was conducted as previously described (*20*). Briefly, an equal volume of clonal population of replication competent vesicular stomatitis virus (rcVSV) with its native glycoprotein replaced by the Wuhan-1 spike protein (rcVSV SARS-CoV-2) (*37*) at an MOI of 0.01 was mixed with serial dilutions of antibodies (5-fold) in cell media to give the desired final antibody concentration. Antibody cocktails were mixed at equal ratios. Virus:antibody mixtures were incubated at 37°C for 1 hour prior to being added to Vero E6 cells. Virus replication was assessed 72hrs after infection in the presence of selected antibodies. Supernatants from the well with the highest concentration of antibody which showed evidence of viral replication (>20% cytopathic effect) was passaged into the subsequent rounds of selection. Infection, monitoring, and collection of supernatants was performed as in the initial round.

Expression and Purification of Soluble Spike Constructs

The soluble S protein mutants were made in a background of the HexaPro stabilization of the spike (*38*), incorporating D614G/K444E/L452R and D614G/K444E/F486S, and the protein was produced as previously described (*39*). One liter of Freestyle cells was transfected with 1mg of SARS-CoV-2 spike DNA premixed with 3mL of Turbo293 Transfection Reagent. The cells were grown for 6 days at 37°C, after which the supernatant was collected by centrifugation and filtration. The supernatant was incubated with nickel resin for 1 hour at room temperature, and then the resin was washed with 1X PBS pH 7.4. The spike was eluted with 20mM HEPES pH 7.5, 200mM NaCl, 300mM imidazole and concentrated before loading onto a Superdex S-200 gel filtration column equilibrated in 1X PBS pH 7.4. The trimer containing peak was collected, concentrated to 1mg/ml, flash frozen in liquid nitrogen, and stored at -80°C until use.

Negative Stain Electron Microscopy

SARS-CoV-2 spike proteins were mixed with CODV fragments at a molar ratio of 1:1.2 and incubated at room temperature for 10 min and then diluted to a concentration of approximately 0.02 mg spike/ml with 10 mM HEPES, pH 7.4, 150 mM NaCl. To make a grid, 4.8-µl of the diluted sample was placed on a freshly glow-discharged carbon-coated copper grid for 15 s. The drop on grid was then

wicked away with filter paper, and the grid was washed and wicked three times. Same volume of 0.75% uranyl formate was added to the grid to negatively stain protein molecules adsorbed to the carbon and immediately wicked away. After three times staining, the grid was allowed to air-dry. Datasets were collected using a Thermo Scientific Talos F200C transmission electron microscope equipped with a Ceta camera at 200 kV. The nominal magnification was 57,000x, corresponding to a pixel size of 2.53 Å, and the defocus was set at -2 μm. Data was collected automatically using EPU. Single particle analysis was performed using CryoSPARC 3.0.

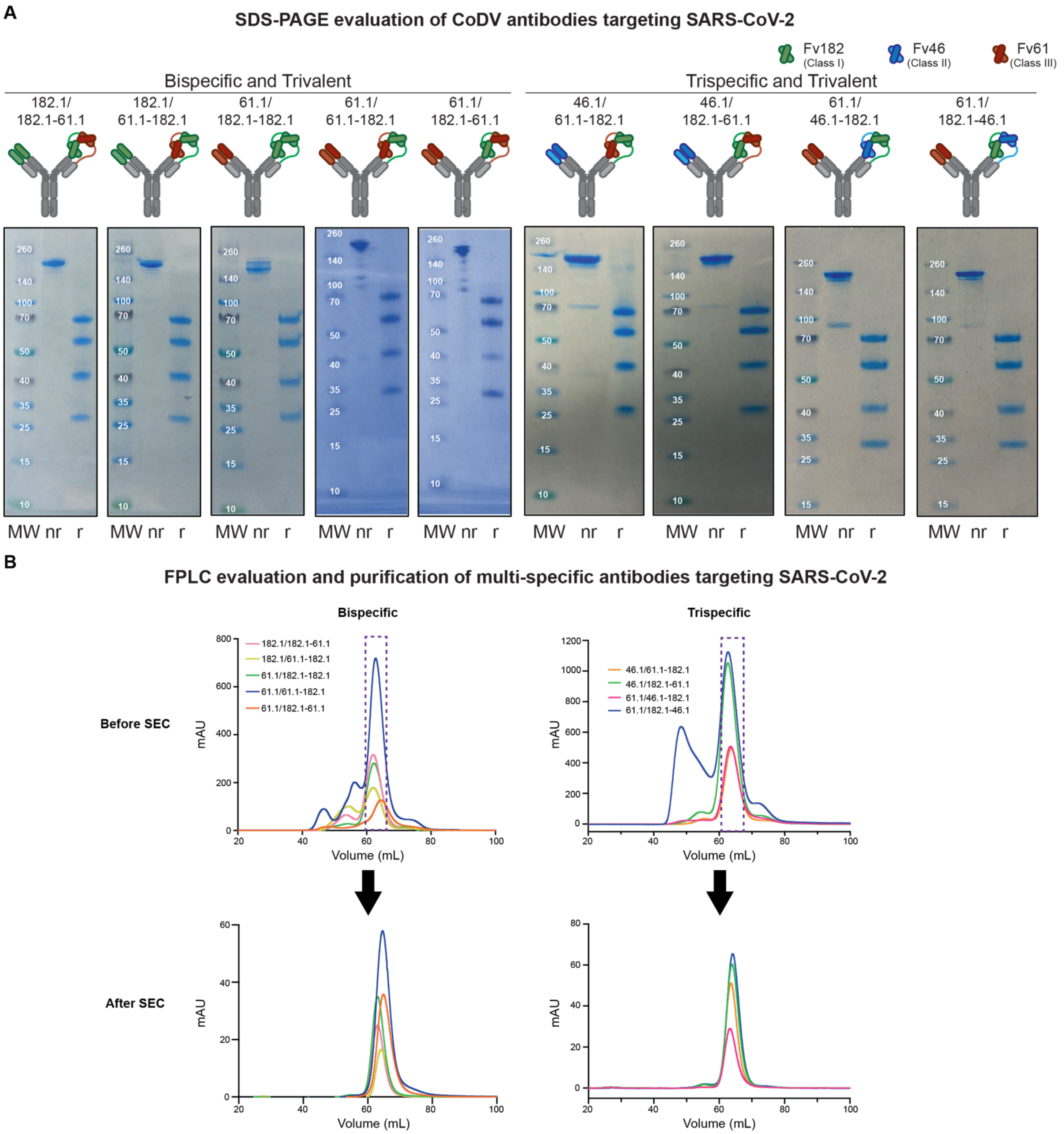

Figure S1. Production and purification of SARS-CoV-2 CODV antibodies
A. Purity of cross-over dual variable (CODV) immunoglobulin antibodies were evaluated under non-reducing (nr) and reducing (r) conditions on a Coomassie SDS-PAGE gel (representative gels shown). **B.** CODV immunoglobulin bispecific and trispecific antibody traces shown before (top row) and after size exclusion chromatography (SEC) (bottom row). The purple box indicates that fractions combined to make final preparations of the indicated multispecific antibodies. The bottom row shows analytic SEC traces for the purified multispecifics. Shown are representative traces from production runs of antibodies. Antibody properties and yields are shown in Table S1.

ID	Molecular Weight (kDa)	Isoelectric Point	Extinction Coefficient. (M^{-1} cm^{-1})	Yield (mg/L)
182.1/182.1-182.1	173.1	8.38	267120	14.5
182.1/182.1-61.1	173.4	8.39	271465	3.94
182.1/61.1-182.1	173.4	8.39	271465	2.6
61.1/182.1-182.1	173.4	8.39	271465	4.37
61.1/61.1-182.1	173.7	7.81	275810	1.6
61.1/182.1-61.1	173.7	7.81	275810	9.6
46.1/61.1-182.1	173.7	8.32	280280	9.24
46.1/182.1-61.1	173.7	8.32	280280	16.41
61.1/46.1-182.1	173.7	8.32	280280	7.94
61.1/182.1-46.1	173.7	8.32	280280	17.72

Table S1. Biochemical properties and production yields following SEC purification for each SARS-CoV-2 CODV antibodies

A

Cross-over dual variable immunoglobulin antibody designs

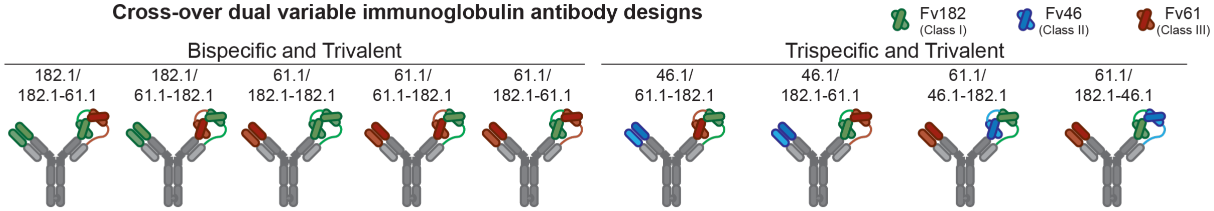

B

Expected binding of Fv components to RBD mutations

	Fv182	Fv46	Fv61
F486S	-	++++	++++
L452R	++++	-	++++
K444E	++++	++++	-

C

Validation of multi-specific Fv binding activity using Fv isolation mutants

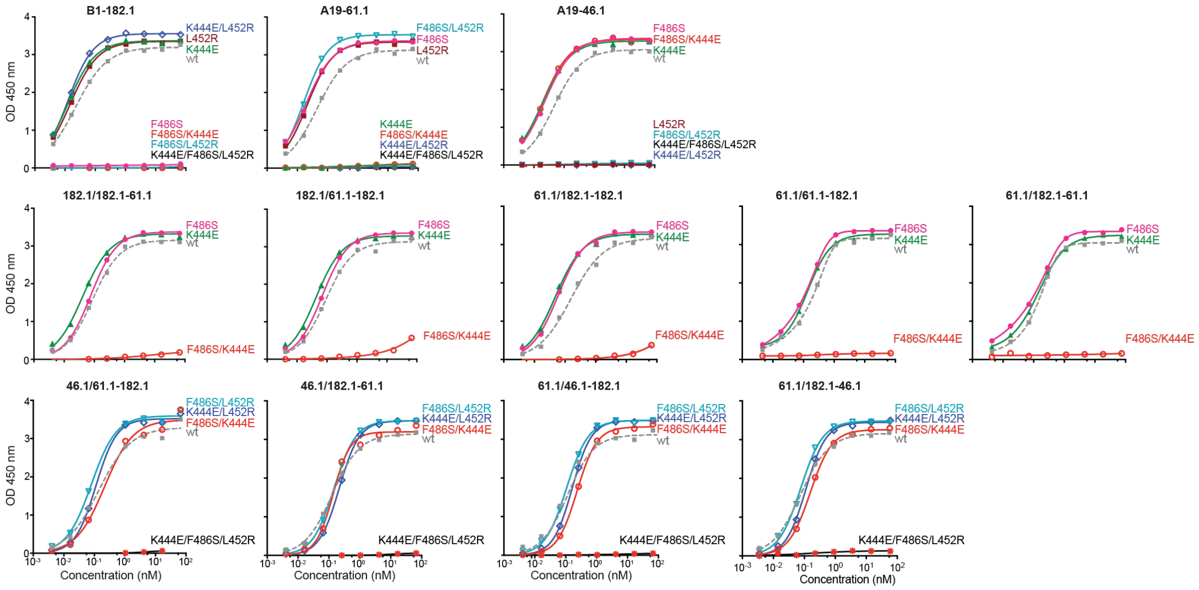

D

Validation of multi-specific neutralization activity using Fv isolation virus mutants

		D614G		B1-182.1 Escape F486S/D614G		A19-46.1 Escape L452R/D614G		A19-61.1 Escape K444E/D614G	
		IC50	IC80	IC50	IC80	IC50	IC80	IC50	IC80
	B1-182.1	15.7	34.9	>67,000	>67,000	25.0	75.8	11.1	29.9
	A19-61.1	44.1	96.5	75.2	166	207	444	>67,000	>67,000
	A19-46.1	113	339	133	390	>67,000	>67,000	472	1504
Bispecific and Trivalent	182.1/182.1-61.1	11.4	36.5	404	1320	24.1	113	7.0	56.1
	182.1/61.1-182.1	15.8	46.8	354	790	38.5	65.2	10.1	35.8
	61.1/182.1-182.1	67.4	128	320	1008	146	312	261	1090
	61.1/61.1-182.1	81.2	108.2	36.7	76.0	174.6	470.3	>67,000	>67,000
	61.1/182.1-61.1	20.2	46.4	29.4	59.0	93.8	230.2	3053.4	8211.7
Trispecific and Trivalent	46.1/61.1-182.1	48.7	119	112	243	201	603	189	487
	46.1/182.1-61.1	31.7	62.9	80.0	271	279	910	46.9	140
	61.1/46.1-182.1	30.8	48.4	68.6	164	233	934	42.0	140
	61.1/182.1-46.1	82.0	183	82.6	212	1010	2097	89.3	356

pM
<10
10-100
100-1000
1000-10,000
10,000-67,000
>67,000

Figure S2. Functional assessment and effect of position of Fv components against RBD knockout mutations

A. Cross-over dual variable (CODV) immunoglobulin antibody designs utilizing variable fragments (Fv) from B1-182.1 (Fv182.1, green), A19-46.1 (Fv46.1, blue) and A19-61.1 (Fv61.1, red). Fc regions contain "knob and hole" feature to increase yield and correct association of heavy chains. Five bispecific trivalent molecules (182.1/182.1-61.1, 182.1/61.1-182.1, 61.1/182.1-182.1, 61.1/61.1-182.1, 61.1/182.1-61.1) and four trispecific trivalent molecules (46.1/61.1-182.1, 46.1/182.1-61.1, 61.1/46.1-182.1, 61.1/182.1-46.1) were designed.

B. Shown are the expected binding of Fv182.1, Fv46.1 and Fv61.1 to RBD proteins containing the indicated Fv isolating mutations, that were selected to knockout binding a single component Fv while leaving binding of the remain Fv components unchanged.

C. Validation of multispecific Fv binding activity using Fv isolating mutations in RBD and ELISA. Shown is ELISA binding for parental component antibodies controls (top row, B1-182.1, A19-46.1, A19-61.1), bispecific antibodies (middle row, 182.1/182.1-61.1, 182.1/61.1-182.1, 61.1/182.1-182.1, 61.1/61.1-182.1, 61.1/182.1-61.1) and trispecific antibodies (bottom row, 46.1/61.1-182.1, 46.1/182.1-61.1, 61.1/46.1-182.1 and 61.1/182.1-46.1) against WA-1 RBD (wt) or RBD proteins containing one or more Fv-specific mutations shown in panel B chosen to interrogate binding of one Fv component at a time in each multispecific. For example, F486S/K444E would show the binding of Fv46 component of trispecific antibodies because it does not allow binding of Fv182. and Fv61.

D. Neutralization of candidate multispecific antibodies against D614G or mutants that target Fv182 (F486S/D614G), Fv46 (L452R/D614G) and Fv61 (K444E/D614G). Neutralization in pM is shown. Ranges are indicated with light blue (>67,000 pM), yellow (>10,000 to ≤67,000 pM), orange (>1000 to ≤10000 pM), red (>100 to ≤1000 pM), maroon (>10 to ≤100 pM), and purple (≤10 pM).
